# A Unique Formulation of Cardioprotective Bio-Actives: An Overview of Their Safety Profile

**DOI:** 10.3390/medicines6040107

**Published:** 2019-10-22

**Authors:** William Salminen, Mayowa Agbaje-Williams, Funmilayo O. Ajayi

**Affiliations:** 1 Camargo Pharmaceutical Services, Cincinnati, OH 45242, USA; wsalminen@camargopharma.com; 2 Clinical Scientist Group LLC, Phoenix, AZ 85286, USA; 3 Annesway Consulting Group LLC, Springboro, OH 45066, USA; ajayifa@yahoo.com

**Keywords:** cardiovascular disease, polyphenols, vascular calcification, oxidative stress, inflammation

## Abstract

The burden of cardiovascular disease (CVD) remains high globally and in the United States despite the availability of pharmaceuticals aimed at its prevention and treatment. An invention by Summit Innovation Labs, which is a formula consisting of a unique blend of select polyphenols (i.e., curcumin, quercetin, resveratrol), vitamin K2 as menaquinone-7, and magnesium, was recently developed to modulate the impact of the specific drivers of CVD, namely, vascular calcification, oxidative stress, and chronic inflammation. The SIL formulation is a dietary supplement that was designed leveraging the more bioavailable forms of ingredients with poor absorption, such as curcumin and quercetin. Each ingredient within the SIL formulation has been shown to contribute to CVD risk reduction by moderating the effect of CVD triggers, thereby providing a holistic prevention strategy for CVD in the healthy population. This review focuses on recently published clinical data to support the safety profile of these ingredients following oral administration. The preponderance of clinical trial data reviewed support the overall safety of the bioactives when used singly or in combination. The most commonly reported adverse effects were generally mild dose-related gastrointestinal disturbances, which may be alleviated with diet in some cases. In light of these, we conclude that the combination of the ingredients in the SIL formulation is reasonably expected to be safe.

## 1. Introduction

Globally, one-third of the more than 54 million deaths in 2013 were attributed to cardiovascular disease (CVD) [1]. Similarly, in the United States, although there was a decrease in CVD mortality between 2000 and 2014, heart disease remains the leading cause of death, with an annual mortality rate of 25% [2,3] despite the availability of myriad pharmacological interventions for the prevention and treatment of heart disease.

Although the mortality rate due to CVD decreased in the past two decades (1990–2013), the global burden of CVD increased roughly during the same period (1990–2010) [1,4,5]. Of note is the increase in the number of CVD deaths, particularly in low to middle income countries, owing to the increasing growth and aging of the global population [1]. More alarming, however, is the deceleration in the rate of CVD mortality decline and the possibility that the decline may stop or even reverse course [6].

Ischemic heart disease has been shown to be the main driver of CVD burden according to the 2010 global burden of disease study [5]. It is caused by the presence of atherosclerosis in the coronary arteries [7]. The development of atherosclerosis in CVD is a multifactorial process thought to be initiated by the presence of oxidized low-density lipoprotein (LDL) particles and their penetration of the endothelial barrier [8]. Oxidized LDL particles signal the recruitment of chemokines and inflammatory mediators, leading to formation of atherosclerotic plaques. In addition to oxidative stress, inflammation and endothelial dysfunction are also recognized pathogenic pathways in CVD development [9,10].

These pathways have been associated with the development of vascular calcification (VC), a major risk factor for atherosclerosis [11,12,13]. VC is distinguished by the presence of calcium deposits in the vasculature leading to reduced elasticity and compliance of the vessel wall, with potential increased risk of death [11,14].

A formulation (SIL) consisting of a unique blend of plant phytonutrients comprising primarily polyphenols (PPs), vitamins, minerals, and other natural bioactive ingredients was recently developed to mitigate oxidative stress and chronic inflammation. PPs have been key components of the human diet for centuries, and within the past couple of decades have been a focus of scientific research geared towards the evaluation of their therapeutic potential against myriad diseases [15]. For instance, diets rich in PPs such as the Mediterranean diet have been linked to a reduced risk of major CV events [16].

The SIL formulation, a dietary supplement developed to modulate the progression of CVD by harnessing the cardiac-health-promoting benefits of PPs, is expected to yield better systemic exposure compared to that following food consumption since culinary preparation such as peeling and boiling is known to significantly reduce the PP content of foods [17,18]. Furthermore, because there are a variety of PPs in food and due to the challenges in the quantitation of individual PPs in certain foods, it is difficult to pinpoint a specific plant source that contains all key PPs that may mitigate CVD [19]. As such, the SIL formulation consists of the following select PPs based on their purported cardioprotective activity in the following daily dose ranges: curcumin 30–150 mg, quercetin 20–100 mg, resveratrol 50–250 mg; as well as vitamin K2 (as menaquinone-7) 30–360 µg, and magnesium 50–300 mg. The dose ranges selected for curcumin and quercetin were reduced by a factor of 5 to 10 given the improved bioavailability conferred by the dose form of these ingredients as discussed below.

PPs are a diverse group of naturally occurring substances that are abundantly present in higher plants and consequently in the human diet [17]. The polyphenolic chemical structure is distinguished by aromatic rings bound to multiple hydroxyl groups [17,19].

PPs are believed to possess cardioprotective effects, in part due to their antioxidant properties conferred by the presence of the phenolic hydroxyl groups, which scavenge reactive oxygen species (ROS) [19,20]. The consumption of a polyphenol-rich diet has been shown to lower LDL oxidation—an initiative step in the development of atherosclerosis [15,21]. In addition, anti-inflammatory properties of PPs are manifested in their modulation of signaling pathways and the adhesive interaction between the vasculature and immune system—a process that is key to plaque formation [15,19].

As will be discussed below, each of the ingredients in the SIL formulation have been shown to impact the effect of one or more of the drivers of CVD, and collectively they provide a holistic means of maintaining optimal cardiovascular health.

The purpose of this article is to review the safety of each of these ingredients following oral administration.

## 2. Literature Review Strategy

First, ingredients were reviewed from a regulatory standpoint by considering Food and Drug Administration (FDA) safety requirements, including the Dietary Supplement Health and Education Act (DSHEA) of 15 October 1994. According to DSHEA, an ingredient marketed prior to 15 October 1994 is *not* considered a new dietary ingredient and does not require the submission of a premarket notification (safety data) to the FDA at least 75 days before marketing the product. To determine if an ingredient is considered an “old dietary” ingredient, the United Natural Products Alliance (UNPA) published a compilation of old dietary ingredients in 2008 to be used as a reference tool. A caveat is that the FDA has not verified the accuracy of the list and as such may reserve the right to require that the company submit safety data. Each ingredient in the SIL formulation was reviewed to see if it is present on the UNPA list and or in currently marketed DSHEA products at the same or higher doses.

Secondly, an in-depth review of the safety and efficacy data on each ingredient in the formulation was performed by searching for literature articles primarily on MEDLINE, PubMed, and Google Scholar; or from internet searches using Google; as well as citations from relevant review articles on each ingredient. Search terms employed included but were not limited to ingredient name, *safety, side effects, clinical trial,* and *adverse effects*. Articles were included if they were published in the last 10 years and contained information on the safety and tolerability of each ingredient when administered orally to human subjects. Select articles published outside of the 10-year window were included if they contained pertinent safety information. A number of clinical studies evaluating the safety and efficacy of these ingredients have been conducted in a variety of indications.

## 3. Curcumin

**Figure 1:**
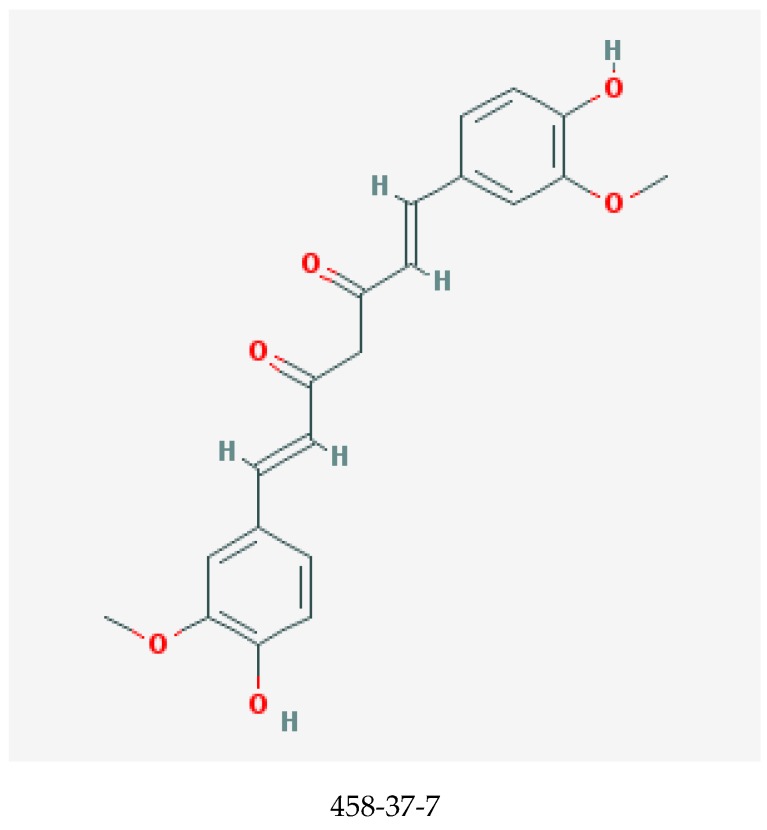
Chemical structure of curcumin with chemical abstracts service registry number (CASRN) [22].

Curcumin, shown in Figure 1, is one of the major and most potent curcuminoids present in the Indian spice turmeric, the powdered rhizome of the plant *Curcuma longa* [23,24]. Other curcuminoids are demethoxycurcumin and bisdemethoxycurcumin [24]. Curcumin can be derived from turmeric by solvent extraction, and the ensuing extract is then purified by crystallization [25].

Curcumin has been used for more than 5000 years in India not only as a food flavoring and coloring agent, but also for its medicinal properties, which include but are not limited to anti-inflammatory, antioxidant, anti-infective, antidepressant, antispasmodic, and wound-healing effects [24]. A preponderance of literature suggests an activity of curcumin against inflammatory disease states such as osteoarthritis [26], autoimmune diseases such as rheumatoid arthritis [27], ulcerative colitis [28], malignant conditions [29,30], diabetes mellitus [31], and cardiovascular diseases [32].

Because of its hydrophobicity, it is highly insoluble with low oral bioavailability [24]. However, it is soluble in dimethylsulfoxide, acetone, ethanol, and oils [24]. A number of measures have been employed to increase the bioavailability of curcumin. These include formulating it as a nanoparticle [33], in liposomes [34], in phospholipids such as lecithin [35], or with piperine, the active ingredient of black pepper [36]. Piperine was found to improve the bioavailability of curcumin by about 2000%, possibly by the inhibition of glucuronidation in the liver and small intestine [36].

In the SIL formulation, curcumin is delivered as theracurmin, a nanoparticle colloidal dispersion synthesized by mixing curcumin powder in gum ghatti solution with water and glycerin and then dispersing with a high-pressure homogenizer [33]. The formulation of curcumin as theracurmin increases the bioavailability of curcumin in rats and humans by at least 27-fold, ensuring efficacy at a relatively low dose within the SIL formulation.

### Safety Profile from Clinical Studies on Curcumin

The studies of curcumin reviewed in Table 1 consisted of a wide variety of subject populations ranging from relatively healthy to critically ill in a primarily adult population. One study of curcumin in a pediatric population was also included to support the wide margin of safety of curcumin across different populations. Additionally, the doses evaluated in the reported studies ranged from 500 to 12,000 mg.

The most common adverse effect of curcumin was gastrointestinal (GI) disturbance, which might be alleviated when taken with food or after a meal, as indicated in the study by Mirzabeigi et al. [32]. One efficacy study in an osteoarthritic population co-administered curcumin with other therapies such as non-steroidal anti-inflammatory drugs (NSAIDs, e.g., naproxen) [38], which are known for causing gastrointestinal (GI) side effects. As such, it is difficult to attribute the adverse event solely to curcumin in such studies. Nonetheless, GI side effects were most often reported in subjects that received curcumin alone.

The greatest exposure to curcumin occurred in a study that administered curcumin in a dose-escalating fashion up to 12,000 mg/day for 3 months in patients with malignancy [40]. That study reported no adverse events up to 8000 mg/day.

Given the long-term history of use and the wealth of data supporting an acceptable safety profile of curcumin at clinical doses up to 8000 mg in both healthy and diseased populations, we conclude that curcumin supplied as theracurmin at doses ranging from 30 to 150 mg per capsule in the SIL formulation is safe even when administered with agents that enhance its bioavailability. In addition, turmeric, the rhizome from which curcumin is derived, is recognized as an FDA approved food additive per 21CFR73.600, suggesting its suitability from a safety perspective for inclusion in the SIL formulation. Furthermore, *Curcuma longa* is listed on the UNPA list.

## 4. Quercetin

**Figure 2:**
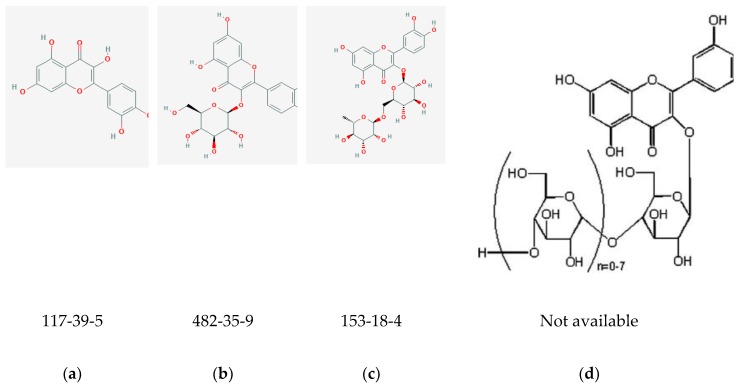
Chemical structures with CASRNs: (**a**) quercetin [42]; (**b**) Isoquercitrin (IQC) [43]; (**c**) Rutin [44]; (**d**) Enzymatically modified isoquercitrin (EMIQ) [45].

Also known as 3,5,7,3′,4′–pentahydroxyflavone, quercetin (Figure 2a) belongs to the flavonol class of flavonoids [46]. It is a phytochemical present in glycosylated form with close to 150 naturally occurring glycosides [47]. Some examples of quercetin glycosides are: rutin or quercetin-3-*O-*β-rutinoside (Figure 2c), isoquercitrin or quercitin-3-*O-*β-glucoside (Figure 2b), and quercitin-3,4′-*O-*β-diglucoside [45]. Quercetin aglycone (or free quercetin) can be derived by extraction of the glycosides from plants followed by hydrolysis [48]. It is abundant in onions, curly kale, leeks, broccoli, blueberries, tea, citrus fruits, grapes, etc. [17,46]. It is considered to be a potent antioxidant due to the presence of hydroxyl groups and double bonds in its structure (Figure 2) [42,46], and has been explored for its therapeutic potential in a variety of indications—particularly cancer and cardiovascular disease [49].

As previously mentioned, atherosclerosis is initiated by infiltration of the endothelial layer by oxidized LDL particles. Other potential contributing processes include reduction in endothelial nitric oxide production and impaired antithrombotic/antiplatelet activity [50,51]. Quercetin has been shown to mitigate the formation of atherosclerotic lesions, reduce markers of oxidative stress (F2-isoprostane), and increase endothelial nitric oxide synthase activity in apolipoprotein E knock-out mice [52]. Quercetin has also been shown in in vitro studies to possess antiplatelet activity and mitigate warfarin-induced vascular calcification [46,53]. This is important given that platelet aggregation and vascular calcifications are believed to contribute to plaque formation [11,54].

In light of these promising therapeutic potentials of quercetin, it is crucial to ensure the consistent delivery of adequate therapeutic levels. The bioavailability of quercetin varies depending on the sugar moiety attached, vehicle for administration, and food source [47]. For example, onions, which contain quercetin glucosides (a simpler sugar) provide higher quercetin bioavailability when compared to apples [55]. Co-administration with high-fat meals is also thought to improve the bioavailability of quercetin [47].

Quercetin’s solubility is poor in water, but is increased in organic solvents or in solutions containing more than 30% ethanol [47]. A wide range of bioavailabilities has been reported for quercetin. This variability may be partly due to the method of analysis utilized, and the food matrix used for administration [56]. In one study conducted in healthy ileostomy subjects administered dried onions (89 mg aglycone), quercetin rutinoside, and quercetin aglycone (both containing 100 mg quercetin aglycone), oral absorption of quercetin was respectively 52%, 17%, and 24% [57]. Hollman et al. (1997) reported the oral bioavailability of quercetin from tea and apples to be 30% that of onions in nine (9) healthy subjects [55]. In general, it is believed that the oral bioavailability of quercetin aglycone is low, and that most of the quercetin in plasma is present in the form of glucuronide and sulfate conjugates [56,58].

Efforts to increase quercetin bioavailability include co-administration with niacin and vitamin C, according to unpublished reports in animal studies by Quercegen Pharma [59]. Some currently marketed formulations consist of enzymatically modified isoquercitrin (EMIQ) and vitamin C. EMIQ or alpha-glycosyl isoquercitrin (Figure 2d), a derivative of rutin, was developed to improve the bioavailability of quercetin. It has been shown to increase plasma levels of quercetin metabolites after oral intake with higher absorption when compared to isoquercitrin and rutin [45]. In rats that were orally administered different quercetin glycosides, the bioavailability of quercetin was found to be 0.8%, 2%, 12%, 30%, and 35% when delivered as rutin, quercetin, isoquercitrin, quercetin-3-*O*-maltoside, and EMIQ, respectively [48].

### Safety Profile from Clinical Studies on Quercetin

Quercetin is one the most studied flavonoids owing to its many potential biological activities [66]. In a detailed review of the safety of quercetin which evaluated results of in vitro, in vivo, preclinical, and clinical studies of quercetin, Harwood et al concluded that the available evidence supports the safety of quercetin for addition to food [56].

Studies of quercetin included in Table 2 spanned the past decade, and no adverse effects of quercetin were reported in all but one study. In that study, of the 1023 subjects enrolled, 667 received 500 or 1000 mg quercetin daily for 12 weeks and nine (9) subjects reported adverse symptoms [59]. Although details of the adverse events were not reported, the authors stated that there was no consistent pattern in the symptoms that were attributed to quercetin. Of note in this study, quercetin was administered with vitamin C and niacin to improve its bioavailability.

Consistent with the detailed review by Harwood et al., which supports the safety of quercetin, and based on the recent studies evaluated in our review spanning doses from 100 to 2000 mg with exposure over a period of 2 to 12 weeks, we conclude that quercetin is safe—especially when administered at the 20 to 100 mg levels in the SIL formulation. In terms of safety considerations from a regulatory perspective, quercetin and rutin, the raw material from which EMIQ is derived, are listed on the UNPA list. In addition, EMIQ has been approved as a food additive in Japan and is present in a number of currently marketed dietary supplement products that can be purchased over the counter and over the internet [47,67].

## 5. Resveratrol

**Figure 3:**
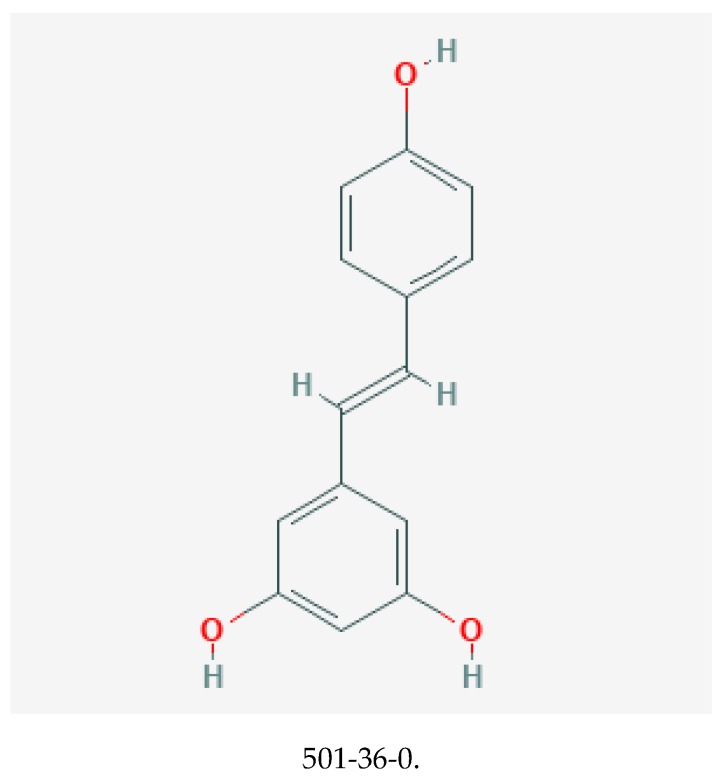
Chemical structure of resveratrol with its CASRN [68].

Resveratrol, with chemical structure 3,5,4′–trihydroxystilbene (illustrated in Figure 3), belongs to the stilbene class of polyphenols [69,70]. It is synthesized by plants in response to stressful stimuli [71]. Resveratrol is present as cis and trans isomers, and its conjugated derivative includes trans-resveratrol-3-*O*-glucoside. Although notably present in red wine, other significant dietary sources include Japanese knotweed (*Polygonum cuspidatum*), grapes, bilberry, and itadori tea [72].

Unlike most of the other polyphenols discussed in this review, resveratrol occurs in minimal concentrations in dietary sources [17,19,69,72]. As such, the delivery of an appreciable amount for potential pharmacological activity might require a nutraceutical formulation which is estimated to deliver up to 83-fold the supply from daily diet [72]. As a nutraceutical, it is usually delivered as 99% purified trans-RSV derived from the rhizome of the Japanese knotweed.

Resveratrol is noteworthy for the French paradox, a term coined to describe the cardioprotective benefit conferred by the French diet which comprises a high amount of red wine when compared to other countries, despite a concomitant high saturated fat intake [73]. The mechanisms proposed include lipid profile improvement and antiplatelet activity, both of which are important for attenuation of atherosclerosis. As a result, resveratrol has been studied extensively for mitigation of CVD risk factors [70,74].

Resveratrol possesses potent antioxidant and anti-inflammatory activity [19]—two biological activities that are germane to the cardioprotective benefits of polyphenols. Its benefits in CVD have been attributed to the reduction of LDL oxidation [75,76], improvement of lipid profile [71,77,78,79], antiplatelet activity [80], as well as antihypertensive and vasodilatory effects [81]. Its vasodilatory effects are believed to occur via improvement of endothelium-dependent vasodilation by increasing nitric oxide bioavailability [82].

As with other ingredients discussed in this review, resveratrol is limited by low bioavailability, which is likely due to its rapid metabolism [70,72]. In a pharmacokinetic study in which twelve (12) healthy males were randomized to receive trans-resveratrol, free resveratrol accounted for approximately 2% of the total concentration in plasma while the glucuronide and sulfate conjugates accounted for >90% [83]. Unlike quercetin, whose absorption is facilitated by high fat intake, resveratrol absorption is impaired with increased fat in the diet [84].

### Safety Profile from Clinical Studies on Resveratrol

As illustrated in Table 3, gastrointestinal adverse events, notably diarrhea, nausea, and abdominal discomfort, were most commonly reported in the studies of resveratrol reviewed. These were usually dose related, occurred more frequently with greater severity at doses above 1 g, and occasionally led to treatment discontinuation. Although some of the studies were limited by small sample sizes, there is a consistent pattern in the reporting of dose-related GI symptoms. The mechanism of GI AE due to resveratrol remains unclear, though the formulation used could be a contributory factor [86].

Resveratrol intake may also lead to a dose-dependent alteration in liver enzymes, particularly AST/ALT and bilirubin. Reduction in liver enzymes was reported more often with doses less than 1 g, while elevations were reported most commonly with doses greater than 1g. The most significant liver enzyme abnormality occurred in a subject receiving 5 g of resveratrol leading to cholestatic liver dysfunction [86]. The subject subsequently discontinued, and liver function returned to normal 8 weeks after drug discontinuation.

Other AEs reported with resveratrol include rash and weight loss. In one study administering 

2 g resveratrol vs. placebo, rash resolved after cessation of resveratrol [87]. Rash did not appear related to dose level. One study reviewed reported weight loss with resveratrol [85]. In this study, subjects were included if they were on stable medications for Alzheimer’s disease management for 4 months. One of the medications used, rivastigmine, has been reported to cause weight loss [91]. For example, rates of weight loss, diarrhea, and nausea for rivastigmine according to the package insert are 18–26%, 7–19%, and 29–47%, respectively [91]. However, these subjects were noted to be stabilized on their respective medications and the article did not identify the specific medications that subjects were on. Of note, the adverse event of weight loss has been previously reported in some animal studies of resveratrol [92,93,94].

Only three studies reviewed reported no adverse events; in these studies, resveratrol was administered at doses significantly less than 1000 mg.

In conclusion, resveratrol is associated with important health benefits as well as dose-related adverse events (i.e., gastrointestinal side effects and alterations in liver enzymes). These were more prevalent at doses greater than 1000 mg per day. Given that the SIL formulation contains 50 to 250 mg resveratrol per day, we anticipate minimal to no adverse events at this dose range. Furthermore, although not listed on the UNPA list, resveratrol is currently marketed in a number of products that can be purchased online as well as in stores.

## 6. Menaquinone-7

**Figure 4:**
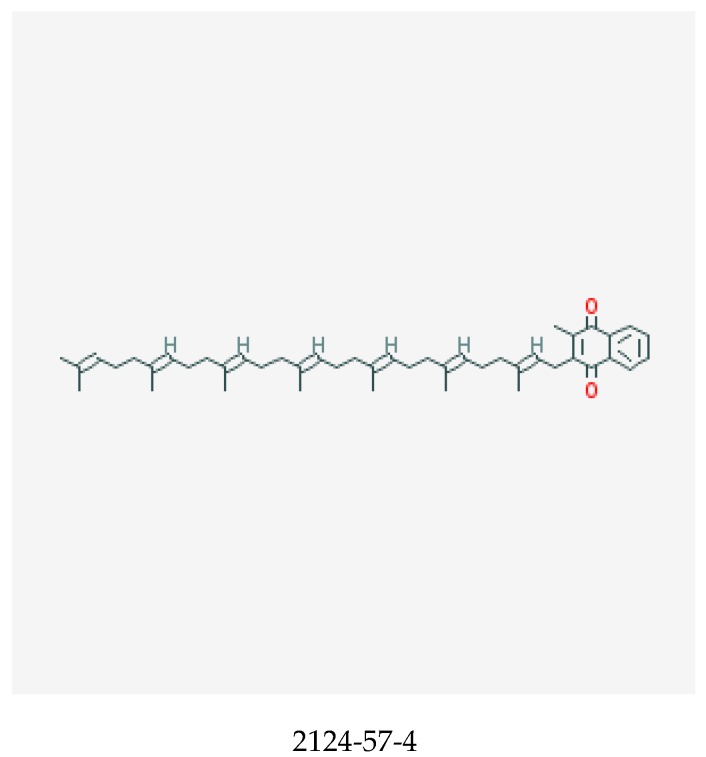
Chemical structure of menaquinone-7 with its CASRN [95].

Vitamin K, shown in Figure 4, is a fat-soluble vitamin that comprises vitamin K1, also known as the phylloquinones and vitamin K2, the menaquinones. Vitamin K compounds possess a common methylated naphthoquinone ring structure, but are distinguished by the length and degree of saturation of the aliphatic side chain at the number 3 position [96,97]. Menaquinones are abbreviated MK-n, with n signifying the number of repeating 5 carbon (prenyl) units [97]. Hence, the longer the side chain, the greater the n. Vitamin K is naturally occurring in plants as phylloquinones, while menaquinones are derived from anaerobic bacterial synthesis in the human gastrointestinal tract [97,98]. Of the 14 known menaquinone compounds (MK-1 to MK-14), MK-4 and MK-7 are the most common [97]. While phylloquinone occurs ubiquitously in green leafy vegetables and some legumes and vegetable oils [97], menaquinones can be derived from meats, eggs, curd, cheese, and fermented soybean, which is a rich source of MK-7 [97,98,99]. Vitamin K is a key cofactor for the enzyme ƴ-glutamyl carboxylase, a process necessary for the activation of hepatic clotting factors [100]—particularly factors II (prothrombin), VII, IX, and X (collectively known as vitamin-K-dependent clotting factors) and proteins C and S, which are endogenous anticoagulants [98]. It is also crucial for production of osteocalcin (OC) in the bone and matrix Gla protein (MGP) in the cartilage and vessel wall.

MGP is necessary for the inhibition of calcification, as was demonstrated by Luo et al. [101]. In that study, MGP-deficient mice were observed to die within two months due to rupture in the abdominal aorta secondary to calcification at multiple sites. In addition, animal and human studies illustrate the association between warfarin—an anticoagulant whose mechanism of action involves inhibition of vitamin-K-dependent clotting factors—and the development of arterial calcification [102,103,104]. This is likely due to the inactivation of MGP, a necessary endogenous calcification inhibitor [105].

The SIL formulation includes vitamin K2 as menaquinone-7 (MK-7) due to studies showing that a diet high in longer-chain menaquinones is associated with decreased risk of coronary heart disease [100,104]. Additionally, a study by Buithuis et al. showed that longer-chain K2 vitamins such as MK-7 are the preferred cofactors for vascular vitamin-K-dependent carboxylase [106]. Further, Schurgers et al. illustrated in healthy volunteers that MK-7 significantly induced the carboxylation of OC and MGP while no such effects were observed for vitamin K1 [107]. A hypothesis for the evaluation of MK-7 for the mitigation of vascular calcification is its longer rate of clearance, as a result of increased lipophilicity and enhanced degree of ƴ-carboxylation [108]. Consequently, a randomized double-blind placebo controlled clinical study evaluating the effects of MK-7 supplementation on the progression of coronary artery calcification is underway.

The absorption of menaquinone from the small intestine was initially thought to be limited due to its hydrophobicity and the lack of bile salts for solubilization and absorption [97]. However, menaquinone has been shown to contribute significantly to hepatic vitamin K content in a similar ratio as phylloquinones. A study of MK-7 in healthy female volunteers administered a single 420-µg dose showed that MK-7 was absorbed to a greater extent and had a longer half-life than the same dose of MK-4 [109]. In another study which compared the absorption of vitamin K1 and MK-7, the bioavailability of MK-7 was found to be 2.5 times that of vitamin K1 expressed as area under the plasma concentration time curve (AUC) over 24 h [107]. In this study, MK-7 remained detectable for at least 4 days.

### Safety Profile from Clinical Studies on Menaquinone-7

It appears that the main safety concern with the use of MK-7 as reviewed in Table 4, is its effect on coagulation parameters, particularly in patients on concomitant anticoagulant—specifically coumarin derivatives. In the study by Theuwissen et al., no coagulation-related AEs were reported in healthy subjects in the range of doses evaluated [110]. The authors surmised that this was to be expected given that the population studied included healthy subjects not on thromboembolic prophylaxis with coumarin derivatives. Conversely, in another study where healthy subjects were treated with acenocoumarol (an anticoagulant coumarin derivative), MK-7 was a more potent inhibitor of anticoagulation when compared to vitamin K1 [107]. A dose of 130 µg/d of MK-7 decreased the international normalized ratio (INR) from 2.0 to 1.5 while a dose of 315 µg/d of vitamin K1 was necessary to achieve a comparable decrease in INR. Based on this observation, the authors proposed an upper safety limit of 50 µg/d of long-chain menaquinone for patients on oral anticoagulant treatment.

The interaction between MK-7 and coumarin anticoagulants is manageable in clinical practice given that patients on warfarin are typically counseled to maintain a consistent intake of a vitamin-K-containing diet, and the dose of warfarin can be adjusted to the patient’s desired vitamin K intake [114].

Overall, MK-7 is expected to be safe in a healthy patient population not anticipated to be on concomitant anticoagulation. Studies reporting adverse events with use of MK-7 had enrolled patient populations with underlying renal dysfunction. In addition, the concern regarding the alteration of coagulation parameters in patients on coumarin derivatives is manageable since these patients are being closely monitored in anticoagulation clinics to ensure therapeutic INR. Finally, the SIL formulation contains MK-7 at doses ranging from 30 to 360 µg, which as reported above are reasonably expected to be safe.

## 7. Magnesium

Magnesium is an essential mineral for a variety of life-sustaining reactions in the body [115]. It is a cofactor for a number of enzymatic reactions which modulate protein synthesis, muscle and nerve function, blood glucose, blood pressure control, etc. It can be derived from many dietary sources. Examples include nuts (almonds), whole grains, green leafy vegetables (e.g., spinach), meats, and dairy [116]. Although magnesium is ubiquitous in the human diet, it can also be derived from dietary supplements and medications such as antacids and laxatives [115].

For medications and/or dietary supplements, magnesium is delivered via different salts with varied bioavailability. Some examples are magnesium oxide, magnesium chloride, and magnesium citrate [115]. A study of magnesium bioavailability from different salts in magnesium-deficient rats showed improved absorption of magnesium from organic salt as compared to inorganic salts [117]. Studies in humans support the improved bioavailability of magnesium when administered as aspartate, lactate, citrate, and chloride as compared to oxide and sulfate [115].

With respect to cardiovascular health, magnesium lowers diastolic blood pressure (albeit to a small extent), modulates vascular tone, and is inversely associated with coronary artery calcification and CV risk factors [118,119,120,121]. When deficient, it is associated with impaired electrical conduction in the cardiac muscle, leading to arrhythmias [122].

Adverse effects of magnesium typically occur from non-food sources used for pharmacologic purposes [122]. The most commonly reported AE from magnesium is diarrhea, hence its use as an osmotic laxative in the form of magnesium hydroxide. In this study, the dose observed to be needed for its laxative effect was 500 mg elemental magnesium which is considerably higher than the recommended dietary allowance (RDA), which ranges from 30–420 mg depending on the gender and age range [115]. When it occurs, diarrhea may be accompanied by nausea and abdominal cramping [122].

Magnesium is an essential mineral, and is currently used in a number of pharmacological products either over the counter or via prescription. The most common AE experienced from magnesium is diarrhea, which as mentioned above is expected to occur in a formulation requiring 500 mg elemental magnesium. In the SIL formulation, the dose of magnesium per capsule ranges from 50 to 300 mg, which is below the RDA, and as such is expected to be safe.

## 8. Safety Profile in Clinical Studies on Combination Ingredients

A clinical study designed to evaluate the safety and efficacy of the SIL formulation is ongoing. Thus far, under the supervision of an integrative medicine physician, 25 subjects with underlying comorbid conditions such as hyperlipidemia and hypertension have taken the SIL formulation. No adverse effects have been reported after 3 months on the SIL formulation. Enrollment remains ongoing and the study will continue for 9 months. This will provide additional safety data on the use of the SIL formulation.

While clinical studies evaluating the safety of the SIL formulation are limited, there are a couple of studies evaluating the combination of one or two of the ingredients together and/or with other ingredients for a number of disease indications.

Torella et al. (2016) assessed a combination of hyaluronic acid, chondroitin sulfate, curcumin, and quercetin for the prevention of recurrent urinary tract infections in postmenopausal women [123]. In this study, the combination was administered orally, and no adverse events were reported. However, the doses evaluated were not specified.

Curcumin (480 mg) and quercetin (20 mg) were administered in combination to five (5) patients with familial adenomatous polyposis three times a day for 9 months to evaluate the effect on adenoma regression [124]. Adverse events reported were mild and included nausea, sour taste after pill ingestion, and loose stools.

A formulation (MPX) containing ellagic acid, quercetin, and resveratrol was evaluated in a Phase I dose escalation study of patients with biochemically recurrent prostate cancer. Patients were administered one (1) 500 mg MPX capsule daily for more than 12 months [125]. Each capsule in the formulation contained 1.2 mg ellagic acid, 9.2 µg quercetin, and 4.4 µg trans-resveratrol. Treatment was initiated at 500 mg/day to a maximum of 4000 mg/day. Treatment-related AEs were mild and included mostly GI AEs in 36% (5/14) of patients. Of these, 4 were in the highest dose group. Seven (7) patients withdrew from study for non-treatment related reasons. The remaining seven remained on the study drug for 21–30+ months.

A pharmacokinetic study examined the steady-state pharmacokinetics and tolerability of trans-resveratrol 2000 mg twice daily with: i) a standard breakfast, ii) a high-fat breakfast, iii) quercetin 500 mg twice daily, and iv) 5% alcohol in eight (8) healthy subjects [126]. Adverse events reported were mild and included diarrhea and transient rash. These are consistent with earlier studies of resveratrol at doses greater than 1 g. Furthermore, quercetin did not alter the pharmacokinetics of resveratrol.

The adverse event patterns reported with these combination studies are consistent with those reported when these ingredients are used individually. They are generally mild and involve the gastrointestinal tract. Supporting the suitability of the use of these ingredients is the fact that some are currently being marketed as single ingredients or in combination, and are available in stores or for online purchase at doses at or well above that in the SIL formulation.

## 9. Drug Interactions

Based on the literature reviewed above, the evidence suggests that each ingredient in the SIL formulation is reasonably expected to be safe at the proposed dose when used singly or in combination. Nonetheless, safety concerns might arise because of the potential for drug interactions when these ingredients are combined based on the purported in vitro activity of some of the ingredients on drug metabolism pathways.

For example, curcuminoids inhibit the activity of a number of cytochrome P450 enzymes (CYPs) as well as p-glycoprotein [127]. Notable CYP enzymes inhibited by curcuminoids include 2C9, 2C19, 2D6, and 3A4. Respectively, these CYP enzymes are known for metabolizing the drugs warfarin, clopidogrel, fluoxetine, and azole antifungals. In the case of warfarin, an anticoagulant with a narrow therapeutic index, there is a theoretical risk of increased toxicity of warfarin leading to increased bleeding. Similarly, in vitro studies report the inhibitory activity of curcumin on p-glycoprotein—an efflux pump responsible for maintaining low cellular concentrations of certain classes of drugs such as chemotherapeutic and immunosuppressant drugs [128,129]. Consequently, there is a potential for elevated systemic levels of p-glycoprotein substrates and ensuing toxicity when administered concomitantly with curcumin. A recent review details the theoretical pharmacokinetic interactions of curcumin [127].

Curcumin possesses many theoretical pharmacologic activities that might potentiate the effect of other prescription and over-the-counter (OTC) drugs with similar activities. For example, curcumin is believed to possess antiplatelet activity due to its ability to inhibit arachidonic acid incorporation into platelets [130] as well as cyclooxygenase (COX) inhibition [131,132]. When administered with other antiplatelet agents such as aspirin, which is readily available over the counter, there may be a theoretical increased risk of bleeding. Therefore, users of such dietary supplements should be counseled on the potential risk of the drug interaction. A similar interaction might be expected when used in conjunction with other NSAIDs due to curcumin’s purported anticoagulant activity [133].

Curcumin has also been reported to exhibit antidepressant activity in a randomized controlled trial of patients with major depressive disorder [134]. In this study, curcumin was administered at a dose of 1000 mg per day for 6 weeks. The authors expressed a concern for the theoretical risk of serotonin syndrome due to the pharmacodynamic interaction between fluoxetine and curcumin, although no signs were observed in the study.

Like curcumin, resveratrol inhibits similar CYP enzymes. A study conducted in 42 healthy volunteers found that resveratrol inhibited CYP 3A4, 2D6, and 2C9 and induced 1A2 after administering 1 g daily for 4 weeks [84]. The authors reported a concern that this could lead to increased toxicity or altered drug efficacy. As is the case with curcumin, there is a theoretical risk of interaction with drugs with narrow indices such as amiodarone and warfarin which are commonly used in patients with CVD [86]. Finally, resveratrol possesses blood pressure lowering and anticoagulant effects, which may lead to additive drug interactions when used concomitantly with drugs possessing similar pharmacological activity [74].

Unlike curcumin’s inhibitory activity on p-glycoprotein, in vitro studies suggest that quercetin induces the activity of p-glycoprotein [135] and CYP3A metabolism of midazolam with repeated exposure in Chinese subjects [136]. Quercetin was also shown to inhibit activity on OATP1B1-mediated transport of pravastatin in Chinese subjects [137].

Overall, the potential for drug–drug interaction is expected to be low because the quantities of curcumin, resveratrol, quercetin, and other ingredients in each dose of SIL are low and within the levels in currently marketed single and combination products.

## 10. Conclusions

Given the increase in CVD burden due to an ever-growing and aging population, the SIL formulation was strategically designed to modulate CVD triggers with the goal of maintaining good cardiovascular health in a relatively healthy population. The SIL formulation consists of select polyphenols (curcumin 30–150 mg, quercetin 20–100 mg, resveratrol 50–250 mg), magnesium 50–300 mg, and menaquinone-7 30–360 µg. These ingredients are currently available in marketed products at doses well above the proposed amounts in the SIL formulation.

As illustrated above, mild gastrointestinal AEs were most commonly reported for the majority of the ingredients. For resveratrol in particular, AEs appeared to be dose dependent with gastrointestinal AEs and alterations in liver enzymes occurred more often at doses above 1 g. Studies on curcumin and quercetin generally reported minimal or no adverse events. For menaquinone-7, the primary concern is alteration of coagulation parameters, which could lead to significant adverse events in patients on concomitant anticoagulants with coumarin derivatives. However, this is not expected to be an issue in otherwise healthy patient populations. Finally, magnesium is widely used over the counter and as a prescription drug, with diarrhea as its well-known adverse event.

Another aspect of this review was a literature evaluation of the potential for drug interactions. The overall conclusion was that although there is a theoretical basis for drug interactions based on the pharmacological profile of these ingredients, the potential for drug interaction is negligible given the low quantities of each ingredient within the SIL formulation.

While the preponderance of clinical trials reviewed in this article support the overall safety of the bioactives within the SIL formulation, a major limitation of this review is the extrapolation of the data on the safety of the individual bioactives to the SIL formulation as a whole. Future studies evaluating safety of the SIL formulation could provide additional insight. Nonetheless, when evaluating the safety of the SIL formulation, it is important to note that the amounts of the bioactives in the SIL formulation are lower than i) the doses evaluated in the clinical trials reviewed and ii) the amount present in currently marketed products containing the same ingredients either singly or in combination. In light of these, we conclude that the combination of ingredients in the SIL formulation is reasonably expected to be safe. Finally, as with any other dietary supplements, the use of the SIL formulation is not expected to replace a healthy diet (e.g., the Mediterranean diet) and the maintenance of cardiac-health-promoting behaviors.

## Figures and Tables

**Table 1:** Summary of Safety Profile of Curcumin.

Reference	Year	Study Design	Duration (months)	N	Study Population	Dose (mg)	Safety Result
[32]	2015	RCT	2	33	Coronary artery disease	2000	GI AEs (diarrhea) reported in two (2) subjects. This resolved with curcumin admin. after meal.
[37]	2017	Open-label	6	57	Healthy subjects with low bone density	1000	No AEs reported.
[38]	2014	RCT	1.5	40	Osteoarthritis of the knee	1500	Mild GI symptoms reported in 37% (7/19) of subjects in curcumin group vs. 19% (4/21) of subjects in placebo group.
[39]	2013	Dose escalation	6	11	Pediatric inflammatory bowel disease	1000–4000	Mild AEs: increased gassiness; not attributed to curcumin.
[40]	2001	Dose escalation	3		Patients with high risk or pre-malignant lesions	500–12,000	No treatment related AEs reported up to 8000 mg/day. 12000 mg/day dose was found intolerable due to the large volume of the dose.
[41]	2006	Dose escalation	Single dose	24	Healthy volunteer	500–12,000	Grade 1 AEs reported in 30% (7/24) subjects: diarrhea, headache, rash, yellow stool. These did not appear to be dose related.

AE: adverse event; GI: gastrointestinal; RCT: randomized controlled trial.

**Table 2:** Summary of Safety Profile of Quercetin.

Reference	Year	Study Design	Duration (months)	N	Study Population	Dose (mg)	Safety Result
[59]	2011	RCT	3	1023	Non-institutionalized subjects	1000–2000	Nine (9) out of 667 subjects assigned to quercetin reported AEs. Authors indicated that there were no differences in GI, skin, allergy or mental symptoms between treatment groups based on symptom log review.
[60]	2007	RCT	1	44	Hypertensive subjects	730	No AEs reported.
[61]	2009	RCT	1.5	93	Overweight or obese subjects	150	No AEs reported.
[62]	2016	RCT	1	22	Pre-hyperuricemic males	500	No AEs reported.
[63]	2016	Dose escalation	1	30	Hepatitis C	250–5000	Mild GI discomfort resolved after taking quercetin after a meal. Overall, quercetin tolerated up to 5000 mg/day without toxicity.
[64]	2011	RCT	2.5	92	Male smokers	100	No AEs reported.
[65]	2008	RCT	0.5	35	Healthy volunteers	50–150	No AEs reported.

AE: adverse event; GI: gastrointestinal; RCT: randomized controlled trial.

**Table 3:** Summary of Safety Profile of Resveratrol.

Reference	Year	Study Design	Duration (months)	N	Study Population	Dose (mg)	Safety Result
[85]	2015	RCT	12 months	119	Mild to moderate Alzheimer’s disease	2000	Nausea, diarrhea, and weight loss were commonly reported AEs which led to treatment discontinuation. Subjects in the placebo group gained 0.54 ± 0.32 kg body weight while resveratrol led to a weight loss of 0.92 ± 4.9 kg (*P* = 0.038).
[86]	2015	Open-label	3 months	24	Friedrich ataxia	1000 (LD) or 5000 (HD)	Adverse GI events such as diarrhea, loose stools, abdominal pain, nausea, and flatulence occurred at a higher rate in the HD group. Liver dysfunction leading to treatment discontinuation occurred in one (1) subject in the HD group. Skin rash was also reported in one (1) subject in the HD group.
[79]	2015	RCT	3 months	60	NAFLD	600	No AEs were reported.
[87]	2014	RCT	1 month	12	Healthy subjects	2000	Mild GI adverse events occurred at a higher rate in the resveratrol group when compared to placebo. One (1) subject on resveratrol experienced pruritic rash which led to study drug discontinuation.
[88]	2014	RCT	3 months	32	Overweight older adults	300 (MD) or 1000 (HD)	Low rate of AEs in treatment groups. Two (2) subjects in HD group withdrew due to GI adverse events. Authors reported non-dose-dependent changes in liver function tests.
[89]	2014	RCT	2 months	20	Overweight or obese men with NAFLD	3000	Mild GI adverse events occurred at a higher rate in subjects on resveratrol. Furthermore, AST and ALT levels were significantly increased.
[90]	2010	Dose escalation	1 month	40	Healthy volunteers	500– 5000	Adverse GI events were most commonly reported at the 2500 mg and 5000 mg dose levels, and these included nausea, flatulence, abdominal discomfort, and diarrhea. The study also noted elevated bilirubin levels at the 500 and 1000 mg dose levels.

AE: adverse event; ALT: alanine aminotransferase; AST: aspartate aminotransferase; GI: gastrointestinal; HD: high dose; LD: low dose; MD: moderate dose; NAFLD: non-alcoholic fatty liver disease; RCT: randomized controlled trial.

**Table 4:** Summary of Safety Profile of Menaquinone-7.

Reference	Year	Study Design	Duration (months)	N	Study Population	Dose (mcg)	Safety Result
[110]	2012	RCT	3	42	Healthy volunteers	10–360	No AEs on thrombin generation observed.
[111]	2017	Single-arm trial	2	60	Renal transplant	360	AEs reported during treatment included increase serum creatinine (unrelated to MK-7) and mild GI symptoms such as nausea, vomiting, diarrhea, and constipation.
[112]	2010	RCT	12	334	Early postmenopausal women	360	No significant AEs were reported. Example of AEs in the MK-7 group included increased nocturnal hot flashes, abdominal pain, and increased palpitations.
[113]	2014	RCT	2	200	Hemodialysis patients	360–1080	Eleven percent (11%) of subjects reported mild GI adverse events. Five (5) deaths occurred in this study; one (1) occurred in the 360 µg group, two (2) in the 720 µg and 1080 µg groups. Four (4) deaths were due to CV event in patients with documented CVD and the other was due to opportunistic infection in an immunocompromised patient. These were thought to be related to the underlying comorbidity.

AE: adverse event; CV: cardiovascular; CVD: cardiovascular disease; GI: gastrointestinal; MK-7: menaquinone-7; RCT: randomized controlled trial.
